# Counterclockwise modular laparoscopic anatomical mesohepatectomy using combined glissonean pedicle (Takasaki approach) and hepatic vein-guided approaches

**DOI:** 10.3389/fonc.2022.1046766

**Published:** 2022-10-26

**Authors:** Zonglei Zhao, Xiaotong Lyu, Xiaoqin Lyu, Lingqun Kong, Baolei Zhao, Wentao Zhu, Qiang Wei, Xutao Lin, Xuefeng Cao, Xingyuan Zhang

**Affiliations:** Department of Hepatobiliary Surgery, Binzhou Medical University Hospital, Binzhou, Shandong Province, China

**Keywords:** hepatic, laparoscopic, anatomical hepatectomy, mesohepatectomy, surgical procedure

## Abstract

**Background:**

Although laparoscopic anatomical hepatectomy (LAH) is widely adopted today, laparoscopic anatomic mesohepatectomy (LAMH) for patients with hepatocellular carcinoma (HCC) remains technically challenging.

**Methods:**

In this study, 6 patients suffering from solitary liver tumors located in the middle lobe of the liver underwent counterclockwise modular LAMH using combined Glissonean pedicle (Takasaki approach) and hepatic vein-guided approaches. In this process, the Glissonean pedicle approach (Takasaki approach) was first used to transect the liver pedicles of segment right anterior (G58) and segment 4 (G4). Second, the hepatic vein-guided approach was performed along the umbilical fissure vein (UFV) to sever the liver parenchyma from the caudal to cranial direction, and the middle hepatic vein (MHV) and anterior fissure vein (AFV) were then disconnected at the root. Last, the hepatic vein-guided approach was once more performed along the ventral side of the right hepatic vein (RHV) to transect the liver parenchyma from the cranial to anterior direction, and the middle lobe of the liver, including the tumor, was removed completely. The entire process was applied in a counterclockwise fashion, and the exposure or transection sequence was G58, and G4, followed by UFV, MHV, AFV, and finally, the liver parenchyma along the ventral side of RHV.

**Results:**

The counterclockwise modular LAMH using combined Glissonean pedicle (Takasaki approach) and hepatic vein-guided approaches was feasible in all 6 cases. The median duration of the operation was 275 ± 35.07 min, and the mean estimated blood loss was 283.33 ml. All of the 6 patients recovered smoothly. The Clavien-Dindo Grade I-II complications rate was up to 33.33%, mainly characterized by postoperative pain and a small amount of ascites. No Clavien-Dindo Grade III-V complications occurred, and the mean postoperative hospital stay was 6.83 ± 1.47 days. Follow-up results showed that the average disease-free survival (DFS) was 12.17 months, and the 21-months OS rate, DFS rate and tumor recurrent rate were 100%, 83.33% and 16.67% respectively.

**Conclusions:**

Counterclockwise modular LAMH using combined Glissonean pedicle (Takasaki approach) and hepatic vein-guided approaches takes the advantages of the two approaches, is a novel protocol for LAMH. It is thought to be technically feasible for patients with a centrally located solitary HCC. The oncologic feasibility of this technique needs to be investigated based on long-term follow-up. A multicenter, large-scale, more careful study is necessary.

## Introduction

Laparoscopic anatomical hepatectomy (LAH), together with open anatomical hepatectomy (OAH), is a hot topic in the liver surgical field and has been demonstrated to be an ideal curative treatment for hepatocellular carcinoma (HCC) (1). Compared to OAH, LAH has the advantages of small trauma, beautiful incision, rapid recovery, a short hospitalization time and light postoperative pain. In addition, LAH versus laparoscopic nonanatomical hepatectomy (LNAH) for selected HCC patients was shown to be associated with increased disease-free survival (DFS), a lower intrahepatic ipsilateral recurrence rate, and comparable long-term overall survival (OS) and postoperative complications (2). Thus, LAH is a popular procedure, and its indications are gradually expanding. However, the optimum approach to complete LAH has not yet been identified.

The Takasaki approach, the extrafascial Glissonean pedicle approach introduced by Takasaki in approximately 1986, is an approach to the pedicles at the hepatic hilus without liver dissection (3). When the hilar plate is pulled down after detaching the liver parenchyma, the right and left Glissonean pedicles can easily be approached (4). Thus, this approach was considered to be a simple and versatile application procedure to carry out LAH (5, 6).

The hepatic vein, a branch of the inferior vena cava running between hepatic segments or lobes and collecting blood from the liver parenchyma, is often used as an anatomical landmark and is continuously exposed on the plane of hepatic disconnection in OAH or LAH (7). Especially in LAH, the operator is often disoriented because of the visual field, so a path guided by the hepatic vein has become valuable (8).

Due to the complex structure of the central region of the liver, which involves the Glissonean pedicles of segment right anterior (G58) and segment 4 (G4), umbilical fissure vein (UFV), middle hepatic vein (MHV), anterior fissure vein (AFV) and the right hepatic vein (RHV), laparoscopic anatomic mesohepatectomy (LAMH) remains technically challenging in the clinic (9). To date, no standard surgical procedure for LAMH has been reported. Herein, we introduce some recent cases of counterclockwise modular LAMH using combined Glissonean pedicle (Takasaki approach) and hepatic vein-guided approaches, which may offer a benefit for difficult procedures.

## Patients and methods

The study was approved by the Institutional Ethics Committee of Binzhou Medical University Hospital. All surgical procedures in the study were performed in accordance with the relevant regulations at our hospital. Informed consent of patients for surgery or invasive treatment was obtained separately before the operation.

### Patient selection

In this study, consecutive patients who underwent LAMH using combined Glissonean pedicle (Takasaki approach) and hepatic vein-guided approaches for HCC from January 1, 2021, to May 31, 2022, at Binzhou Medical University Hospital were included. Patients with benign tumors or other types of malignant tumors and patients who underwent LNAH were excluded.

### Perioperative care

All patients received preoperative laboratory tests, including routine blood, blood biochemical index, blood clotting, hepatitis B virus and HBV-DNA tests, if necessary. Child−Pugh classification and indocyanine green retention rate at 15 minutes (ICG-R15) were required, as patients suffering from LAMH are at risk of acute liver failure (ALF) after major hepatectomy. Only patients with a Child−Pugh grade A or B, estimated remnant liver volume >40%, and ICG-R15 <25% were allowed to undergo the protocol. A three-dimensional (3D) reconstruction model of the liver for each patient was also built by the IQQA-Liver system (EDDA Company, USA) using the preoperative computed tomography (CT) or magnetic resonance imaging (MRI) image, which could vividly visualize the target Glissonean pedicle (G58 and G4), main hepatic vein (UFV, MHV and AFV) and its important branches. Moreover, the system helps to measure the residual liver volume and the standard liver volume.

All patients received general anesthesia with a central venous pressure controlled at 2-5 cm H_2_O. An experienced surgical team, including 3-4 surgeons, 1-2 anesthesiologists, and 1-2 instrument nurses, completed the operation together with or without indocyanine green fluorescence staining.

Postoperative management was relatively simple, including hepatinica treatment, rehydration, infection prevention, etc. Chest and abdominal CTs were required to be reviewed to assess for the presence of reactive pleural effusion and peritoneal encapsulated effusion after the operation.

### Surgical procedure

All LAMH procedures were performed by the same surgical team. During the protocol, patients were placed in the supine position with legs apart under intravenous and inhalational anesthesia. Double main operator mode was performed, of which one main operator stood on the right side of the patient, and another main operator stood on the left, while the assistant holding the scope stood between the patient’s legs. The pneumoperitoneum pressure was maintained at 10-14 mmHg, and the central venous pressure was maintained at 2-5 cm H_2_O. Five ports were routinely needed, including one 10-mm observation port, two 12-mm operating ports, and two 5-mm assistant ports.

During the protocol, to avoid the spreading of malignant cells, the liver was freed from the ligamentum teres hepatis and falciform ligament without hard compression. Cholecystectomy was performed routinely, or the gallbladder was suspended after disconnecting the gallbladder duct and artery if the bottom or body was invaded by HCC, avoiding direct contact with the tumor. The Pringle maneuver was conducted extracorporeally and intermittently during the transection of the liver parenchyma with the “15-min clamping and 5-min release” principle.

**Figure 1:**
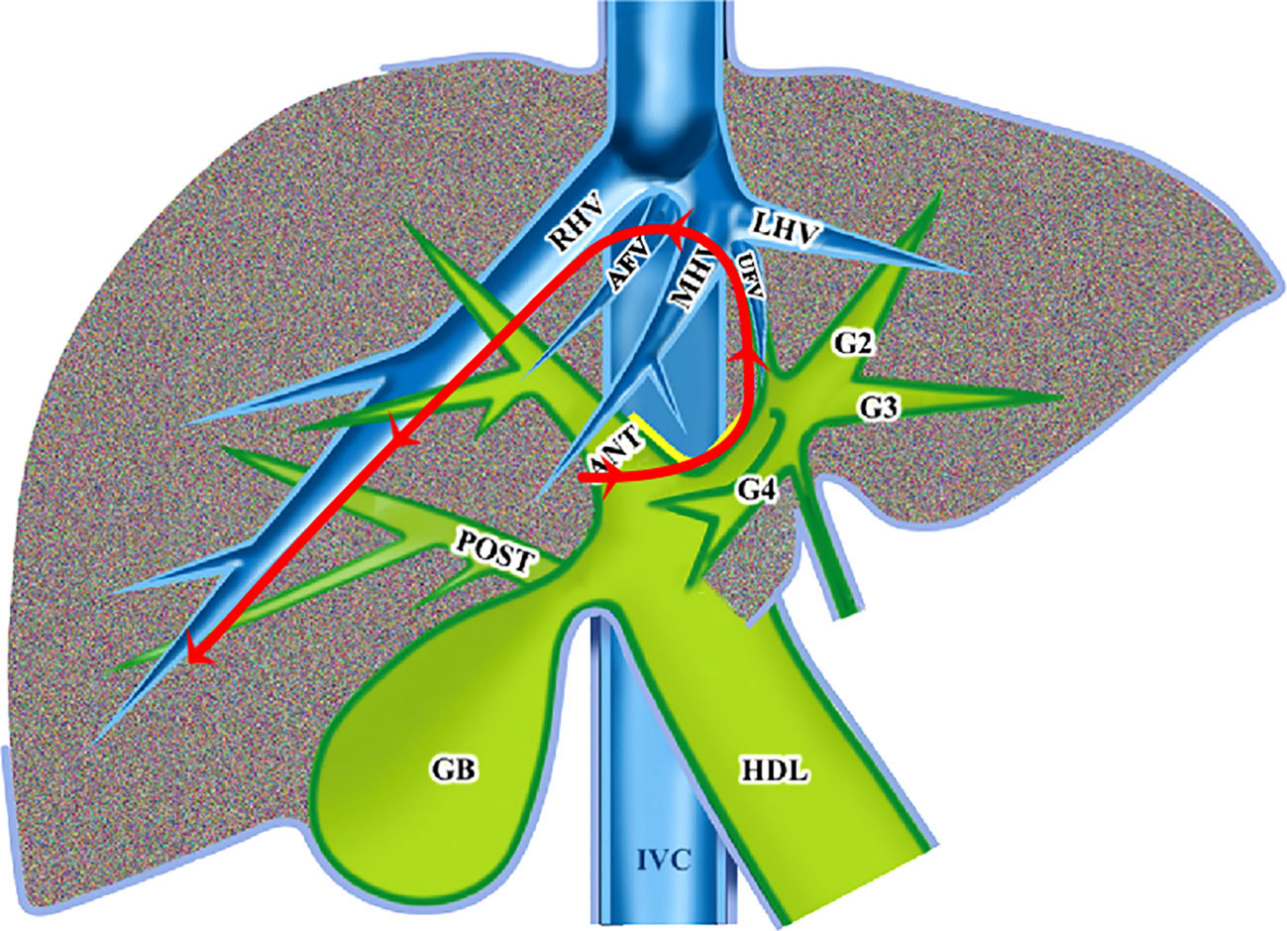
Program diagram of counterclockwise modular LAMH using combined Glissonean pedicle (Takasaki approach) and hepatic vein-guided approaches.

The modular procedure began with the handling of the G58 using the Takasaki approach and hilar plate descending technique, and the entire process was then applied in a counterclockwise sequence. After approaching the target pedicles of G58, clips were used to test the clamp, and the Glissonean pedicles of G4 were then approached by dissecting the umbilical fissure. Usually, 3-4 branches of G4a and G4b were disconnected, and the UFV was exposed during this process. Negative fluorescent staining, through the injection of indocyanine green (ICG) (1 ml, 5 mg/L) from peripheral veins, helped to accurately disconnect the liver parenchyma, and blood flow into the middle liver had, in theory, been completely controlled at this time. If the demarcation line of the ICG fluorescence-negative regions, normally consistent with the ischemic line, was satisfactory, the target pedicles of G58 could be transected subsequently with an Endo&GIA (Johnson & Johnson Company, USA). The hepatic vein-guided approach was first performed along the trunk of the UFV to transect the liver parenchyma from the caudal to cranial direction, and the second porta hepatis was then easily reached. The MHV was subsequently transected at the root using Endo&GIA, followed by the transection of the AFV in the same manner. Both these procedures were completed by the first main operator standing on the right side of the patient. Another main operator on the left then completed subsequent procedures along the ventral side of the RHV to transect the liver parenchyma from the cranial to the caudal direction using a hepatic vein-guided approach, and the whole RHV trunk was exposed at the surgical plane. So far, the whole protocol has been completed, and the middle lobe of the liver, including the tumor, was removed completely. Concrete process was displayed schematically in **Figure 1**.

### Data collection

All data were collected from our clinical database, including age, sex, body mass index (BMI), hepatitis B virus status, Child−Pugh class, ICG-R15, duration of operation, estimated intraoperative blood loss, and times of the Pringle maneuver; postoperative outcomes, such as levels of alanine aminotransferase (ALT) and glutamic oxaloacetic transaminase (AST) on the first day after the operation (POD1); postoperative length of hospital stay; and postoperative complications, classified according to the Clavien–Dindo classification, including abdominal dropsy, pleural effusion, postoperative intra-abdominal hemorrhage, bile leakage, and intra-abdominal infections (IAIs) were also collected. The duration of the operation, estimated intraoperative blood loss, and times of Pringle maneuver data were obtained from the anesthesia records. First-day levels of ALT and AST, the length of postoperative hospital stay, postoperative intra-abdominal hemorrhage data, bile leakage data, and IAI data were obtained from our clinical records. Follow-up was standardized using telephone and outpatient follow-up, and the MRI of upper abdomen was necessary in each outpatient follow-up to assess the tumor prognosis. The overall survival (OS) rate, tumor recurrent rate, disease-free survival (DFS) rate and the average DFS were recorded respectively.

### Statistical analysis

Continuous variables are expressed as the mean and standard deviation (SD). The continuous and categorical variables were compared using ANOVA and Chi-squared tests, respectively. All analyses were performed with the Statistical Package for Social Sciences (SPSS) version 24.0 software (IBM Co, Armonk, NY, USA). Survival was evaluated using the Kaplan-Meier method.

## Results

### Patient characteristics

Seven LAMHs were performed in this study. One patient was excluded because she had no background of hepatitis B and cirrhosis, and the postoperative pathological examination revealed intrahepatic cholangiocarcinoma (ICC) rather than HCC. Therefore, 6 patients (4 males, 2 females) with a mean age of 54.50 years were included in the study and underwent the counterclockwise modular LAMH using combined Glissonean pedicle (Takasaki approach) and hepatic vein-guided approaches. The mean BMI of the 6 patients was 25.50 kg/m^2^. All 6 patients had a history of hepatitis B virus and cirrhosis, but their Child−Pugh stages were classified as A or B, and the mean ICG-R15 (%) was 6.19. The demographic characteristics of the 6 patients are displayed in **Table 1**.

**Table 1:** Demographic characteristics of the included patients.

Parameter	*N=6*
Age (years)	54.50 ± 10.75
Gender	
Male	4 (66.67%)
Female	2 (33.33%)
BMI	25.50 ± 1.70
Underlying liver disease	
Hepatitis B	6 (100%)
Cirrhosis	6 (100%)
Stages of Child-Pugh	
A	4 (66.67%)
B	2 (33.33%)
ICG-R15 (%)	6.19 ± 1.70
Preoperative ALT (U/L)	49.25 ± 39.15
Preoperative AST (U/L)	52.10 ± 35.02
Tumor size in CT/MRI (cm)	5.17 ± 0.72

### Surgical outcomes

All 6 counterclockwise modular LAMHs using combined Glissonean pedicle (Takasaki approach) and hepatic vein-guided approaches went smoothly. The median duration of the operation was 275 ± 35.07 min, and the mean estimated blood loss was 283.33 ml. The overall postoperative recovery was relatively uneventful. The ALT and AST levels, had no significant elevation, were 285.10 ± 95.36 U/L (Normal Range: 15 - 40 U/L) and 265.57 ± 66.74 U/L (Normal Range: 9 - 50 U/L) respectively on POD1. The Clavien-Dindo Grade I-II complications rate was up to 33.33%, mainly characterized by postoperative pain and a small amount of ascites. No Clavien-Dindo Grade III-V complications, such as postoperative intra-abdominal hemorrhage, bile leakage, or IAI, even dead, occurred, and the mean postoperative hospital stay was 6.83 ± 1.47 days.

Follow-up checkups for 4 - 21 months (mean, 12.5 months). All patients survived in the last follow-up, but one case relapsed at the 13th month after operation, and transcatheter hepatic artery chemoembolization (TACE) followed by target therapy and immunotherapy were received then. The average DFS was 12.17 months, and the 21-months OS rate, DFS rate and tumor recurrent rate were 100%, 83.33% and 16.67% respectively. Details of the surgical outcomes are displayed in **Table 2**.

**Table 2:** Details of surgical outcomes of the included patients.

Parameter	*N=6*
Estimated blood loss (ml)	283.33 ± 103.28
Patients transfused in PD	0
duration of operation (min)	275 ± 35.07
Times of Pringle maneuver	4.17 ± 1.17
ALT on POD1 (U/L)	285.10 ± 95.36
AST on POD1 (U/L)	265.57 ± 66.74
Postoperative hospital stay	6.83 ± 1.47
*Complications*	*2 (33.33%)*
* Clavien-Dindo Grade I-II*	*2 (33.33%)*
postoperative pain	1 (16.67%)
ascites	1 (16.67%)
* Clavien-Dindo Grade III-V*	*0*
postoperative intra-abdominal hemorrhage	0
bile leakage	0
IAI	0
OS rate	6 (100%)
DFS rate	5 (83.33%)
Tumor recurrent rate	1 (16.67%)
average DFS (month)	12.17

## Discussion

Currently, laparoscopic mesohepatectomy, especially LAMH, remains a challenging procedure. Although the selection of appropriate patients and detailed preoperative evaluations, such as 3D visual structure reconstruction, help to ensure the success of the operation, an increased number of vessels in the middle hepatic lobe, multiple variations in the vessel course between the anterior and posterior regions, and a relatively narrow operating space under the diaphragm are all unfavorable factors restricting the protocol. Simplifying these complications is a critical topic faced by hepatobiliary surgeons.

In 1985, Couinaud published a report on left hepatectomy with the extrafascial approach in *Surgery* (10), which is the predecessor and the earliest application of the Takasaki approach. In 1986, Takasaki presented a novel liver segmentation approach that divided the liver into three main parts and a caudate area according to the ramification of the Glissonean pedicles. On this basis, he published the extrafascial approach (Takasaki approach) in Japanese and reported that it can be used not only for the main portal pedicle but also for the sectional portal and segmental pedicles in the left and right liver (3, 4, 11–13). Therefore, various types of AH can be carried out with the Takasaki approach. Since the 1980s, this approach has provided new knowledge of surgical anatomy and techniques, and various types of AH have been safely achieved by the Takasaki approach (6, 14, 15). Furthermore, the Takasaki approach, used in AH or LAH, has also been demonstrated to have a potential oncology clinical benefit (16, 17).

Nevertheless, the Takasaki approach without liver dissection could be better utilized. To this end, the existence of Laennec’s capsule needs to be recognized. Laennec’s capsule can be separated from Glisson’s capsule outside and inside the liver, including the main portal pedicles as well as the sectional and segmental pedicles, and can be approached at the hepatic hilus (18). Because of the existence of Laennec’s capsule, the Glissonean pedicles can be easily and safely separated by blunt separation rather than by an incision of the liver parenchyma, thus facilitating the Takasaki approach in AH or LAH. Many related studies have also demonstrated that Laennec’s approach based on Laennec’s capsule can contribute to the standardization of the surgical technique for LAH and bring innovations that facilitate safe and effective liver resection under laparoscopy (19–21).

As described previously, the hepatic vein is the boundary of the Couinuad segment; thus, it is often used as an anatomical landmark in OAH or LAH. Continuous exposure on the plane of hepatic disconnection is usually regarded as a successful sign for OAH or LAH. However, its isolation and exposure is a high-risk procedure, and a slight mistake might lead to massive bleeding or other serious consequences and require converting to an open procedure. In current practice, the hepatic vein approach can be subdivided into the caudal approach, caudal-dorsal approach, cranial-ventral approach and cranial-dorsal approach according to different target veins (22–24). The caudal approach or caudal-dorsal approach used in the dissection of the liver parenchyma has several limitations; for instance, it is prone to lacerate the target vein; thus, the “tenting sign of the hepatic vein” helps to identify the running of the main trunk of the hepatic vein (8), and the approach should be performed by experienced surgeons at experienced centers for well-selected patients (23). In the cranial-ventral approach or cranial-dorsal approach, the hepatic parenchyma is transected from the root of the target hepatic vein toward its distal branches. Its primary advantage is that the liver resection plane can be clearly and safely exposed from the cranial and dorsal sides, and the branches of the target hepatic vein can then be managed separately; thus, it is regarded as a feasible and effective technique during laparoscopic hepatectomy, contributing to the process of LAH by fully exposing and protecting the hepatic veins (24, 25).

In the traditional LAMH, although the Takasaki approach was possibly used, restricted to the standing position of the surgeon, only the caudal hepatic vein-guided approach could be used when completing the right plane, meaning that the RHV would be isolation and exposure from the distal branches to the trunk, which was prone to get lost in the disconnection and lacerate the target vein, leading to massive bleeding or other serious consequences. Different from the traditional LAMH, the protocol in our study takes advantage of both the Takasaki approach and the hepatic vein-guided approach. Because squeezing liver tissue during the operation could release cancer cells, the Glissonean pedicle was implemented as a priority strategy, and ligature and transection were performed at the root of G58 and G4 first. Then, considering that the vasculature between the anterior and posterior regions of the liver varies greatly, we were not in a hurry to transect the liver parenchyma between them but instead completed the left plane of the LAMH based on the characteristics of relatively fixed and less variable nature of G4. After the disconnection of the MHV and AFV, the root of the RHV was easily exposed. Another main surgeon on the left side of the patient subsequently used a cranial approach along the ventral side of the RHV, avoiding the limitation of narrow spaces under the diaphragm when using the caudal approach, under conditions of which the RHV would be fully and safely exposed and protected. Moreover, the RHV-guided approach could effectively avoid the interference of vascular variation between the anterior and posterior regions of the liver and achieve true LAMH. No significant elevations in ALT and/or AST levels occurred on the first day after the operation, which also supported the changes after LAH.

In this study, there was still one patient relapsed at the 13th month after operation. The recurrent tumors were located both in the left lobe and the right posterior lobe of the liver. Thus, a TACE followed by target therapy and immunotherapy were performed. Fortunately, the tumors had no further progress and the patient survived with tumor in the last follow-up. Review the preoperative tumor staging of the patient, although the size of tumor is not massive, the close relationship with the G58, may be the cause of such poor prognosis.

However, this study remains subject to several limitations. First, this is a single-center study with a small sample size and no comparative sequence, which may bias the conclusion. Second, it lacks long-term follow-up to verify whether the procedure has value. Thus, this maneuver should continue to be explored.

In conclusion, counterclockwise modular LAMH using combined Glissonean pedicle (Takasaki approach) and hepatic vein-guided approaches is thought to be technically feasible for patients with a centrally located solitary HCC. The oncologic feasibility of this technique needs to be investigated based on long-term follow-up. A multicenter, large-scale, more careful study is necessary.

## Data availability statement

The original contributions presented in the study are included in the article/supplementary material. Further inquiries can be directed to the corresponding author.

## Ethics statement

The studies involving human participants were reviewed and approved by Institutional Ethics Committee of Binzhou Medical University Hospital, Shandong Province, China. The patients/participants provided their written informed consent to participate in this study. Written informed consent was obtained from the individual(s) for the publication of any potentially identifiable images or data included in this article.

## Author contributions

XC and ZZ contributed equally to this work. They are the guarantors of the manuscript and contributed to conception and design of the study, acquisition and analysis of data, and writing and revision of the manuscript. XTL, XQL, LK, and XZ contributed to pathological experiment, acquisition of the data, and writing and revision of the manuscript. BZ, WZ, QW and XL contributed to data analysis and revision of the manuscript. All authors contributed to the article and approved the submitted version.

## Funding

This research was supported by the Natural Science Foundation of Shandong Province (No. ZR2021MH173), and the Project of Medical and Health Technology Development Program in Shandong Province (No. 202104081018).

## Conflict of interest

The authors declare that the research was conducted in the absence of any commercial or financial relationships that could be construed as a potential conflict of interest.

## Publisher’s note

All claims expressed in this article are solely those of the authors and do not necessarily represent those of their affiliated organizations, or those of the publisher, the editors and the reviewers. Any product that may be evaluated in this article, or claim that may be made by its manufacturer, is not guaranteed or endorsed by the publisher.
